# Staff-mediated doll therapy in Chinese residential dementia care: an exploratory pilot and feasibility mixed-methods cluster randomized clinical trial

**DOI:** 10.3389/fpubh.2026.1935007

**Published:** 2026-09-03

**Authors:** Zhenti Cui, Rongrong Guan, Tianrui Xue, Yongchang Sun, Maw Pin Tan, Mas Ayu Said, Yingdong Cao

**Affiliations:** 1 School of Medicine, Sias University, Zhengzhou, Henan, China; 2 Department of Social and Preventive Medicine, Universiti Malaya, Kuala Lumpur, Malaysia; 3 School of Art and Design, Zhengzhou University of Industrial Technology, Zhengzhou, Henan, China; 4 Division of Geriatric Medicine, Department of Medicine, Faculty of Medicine, Universiti Malaya, Kuala Lumpur, Malaysia

**Keywords:** China, cluster randomized clinical trial, dementia, doll therapy, mixed methods, neuropsychiatric symptoms, nursing homes, psychotropic medication

## Abstract

**Background:**

Doll therapy is a person-centered intervention for behavioral and psychological symptoms of dementia, but evidence from Chinese nursing homes and information about implementation, medication use, and ethical acceptability remain limited.

**Methods:**

We conducted an exploratory pilot and feasibility mixed-methods cluster-randomized clinical trial in six nursing homes in Zhengzhou, China. Homes were randomized 1:1 to a staff-mediated doll-therapy care package plus routine care or routine care alone. Residents with dementia were assessed at baseline, 6 weeks, and 12 weeks. Registered primary outcomes were class-specific standardized daily psychotropic doses; NPI-12 was the main proximal clinical outcome. Feasibility, fidelity, safety, and interviews with intervention-home staff and family caregivers were also evaluated. Marginal models used nursing home as the top-level cluster and the Mancl-DeRouen small-sample variance correction.

**Results:**

Among 185 residents screened, 142 were enrolled, and 130 (91.5%) completed 12-week follow-up. Sixty-eight of 71 intervention participants (95.8%) attended at least 6 of 8 sessions; mean fidelity was 94.1%. The intervention-control difference in NPI-12 change was −3.61 points at 6 weeks (95% confidence interval [CI], −5.86 to −1.36) and −4.40 at 12 weeks (95% CI, −6.50 to −2.30). The Barthel difference was 3.96 points at 6 weeks (95% CI, 1.06 to 6.86) and 3.07 at 12 weeks (95% CI, −2.33 to 8.47). All medication-dose intervals showed no difference. Six possible or probable related events were mild or moderate; no serious event was related. Interviews with 15 staff and 9 family caregivers indicated engagement for some residents, absent or adverse responses for others, and the importance of training, non-deceptive introductions, prompt respect for refusal, and operational ownership.

**Conclusion:**

The care package was feasible and produced a preliminary behavioral signal, but medication effects were imprecise, and functional improvement was not sustained. A larger cluster trial with an attention-control condition is required.

**Trial registration:**

ClinicalTrials.gov, NCT06506487; https://clinicaltrials.gov/study/NCT06506487.

## Introduction

Dementia is a major and growing public-health challenge. Its prevalence rises sharply with age, and population aging has increased the number of people who require long-term assistance with cognition, behavior, mobility, and daily living (1, 2). The Neuropsychiatric Inventory describes domains including agitation, depression, anxiety, apathy, disinhibition, sleep disturbance, and psychotic symptoms that frequently coexist with cognitive decline (3). These symptoms may be frightening for residents, distressing for families and staff, and difficult to manage when staffing, specialist access, and continuity of medical review are limited. Pharmacological treatment is sometimes necessary, but average benefits for behavioral symptoms are modest and vary by indication (4). China faces this challenge at considerable scale because of rapid demographic aging, a large population living with dementia, and marked variation in diagnostic, community, and long-term care resources (5).

Antipsychotics can be associated with sedation, cerebrovascular events, pneumonia, extrapyramidal symptoms, and mortality in frail people with dementia (6), while psychotropic burden more generally is associated with falls in nursing-home populations (7). Guidelines and reviews consequently recommend non-pharmacological, person-centered approaches as core components of dementia care rather than as optional activities (8, 9). However, a behavioral intervention does not automatically lead to a reduction in medication. Prescribing decisions are made by clinicians and influenced by medication review schedules, acute illness, family preferences, staff observations, institutional routines, and the availability of alternatives. Medication outcomes in pragmatic psychosocial trials may therefore be best interpreted as indirect stewardship signals alongside more proximal measures of resident behavior and wellbeing.

Doll therapy is one person-centered approach used in dementia care. A resident is offered a lifelike doll as an optional object for comfort, activity, reminiscence, or caregiving behavior. Earlier reviews described possible improvements in agitation, mood, communication, and engagement, and also emphasized small samples, inconsistent protocols, weak controls, and limited implementation reporting (10, 11). A recent systematic review and meta-analysis found favorable pooled effects for behavioral and psychological outcomes, including agitation, but no clear cognitive improvement (12). The theoretical rationale is often linked to attachment and caregiving systems. Attachment theory proposes that security-seeking and affect regulation become especially salient under uncertainty or loss (13), and these concepts have been applied to dementia-related searching for familiar roles and figures (14). For some residents, holding, comforting, or grooming a doll may support continuity of identity and purposeful interaction. For others, the object may be irrelevant, unwelcome, or distressing. In China, these responses also occur within a care context in which biomedical treatment, family expectations, institutional care, and traditional Chinese medicine may coexist (15).

Doll therapy remains ethically contested. Potential harms include infantilizing an adult, inviting deception, overriding refusal, creating excessive attachment, or using an object as a substitute for human attention (16, 17). Ethical acceptability depends not only on the doll but also on how it is introduced, whether participation is genuinely optional, how verbal and non-verbal dissent are interpreted, and whether staff can adapt or stop the activity promptly. These considerations make implementation outcomes, fidelity, safety, and accounts from staff and families scientifically central.

We therefore conducted an exploratory pilot and feasibility mixed-methods cluster randomized clinical trial of a structured, staff-mediated doll-therapy care package plus routine care vs. routine care alone in Chinese nursing homes. The registered primary aim was to estimate class-specific changes in standardized psychotropic medication dose. NPI-12 was designated as the main proximal clinical outcome, with daily living, cognitive-enhancer use, traditional Chinese medicine, engagement, feasibility, fidelity, and safety as secondary or contextual outcomes. Interviews examined acceptability, cultural fit, training, ethics, implementation burden, and alternative explanations. With six randomized homes, the trial was designed to estimate feasibility and identify preliminary signals rather than to provide definitive evidence of efficacy (18).

## Methods

### Design and reporting

This was an explanatory sequential mixed-methods study comprising a pragmatic, parallel-group, 1:1 exploratory pilot and feasibility cluster-randomized clinical trial followed by a qualitative descriptive study in intervention homes. The nursing home was the unit of randomization because staff routines, activity spaces, prescribing processes, and communication among residents and caregivers made individual randomization vulnerable to contamination. Quantitative assessments occurred at baseline, 6 weeks, and 12 weeks. Qualitative interviews were conducted after participants and staff had sufficient experience with the intervention to interpret implementation and observed responses. The protocol, outcome hierarchy, and analysis principles were published prospectively (18), and the intervention was described according to the Template for Intervention Description and Replication (TIDieR) (19).

### Setting, recruitment, and participants

The trial was conducted in six registered nursing homes in Zhengzhou, Henan Province, China. Site preparation and staff training began in 2024; resident screening, consent, baseline recruitment, intervention delivery, and follow-up were completed during 2024–2025.

Potentially eligible residents were identified from routine records. Eligibility required age 65 years or older, residence in a participating home, documented dementia, ability to recognize a doll-sized object, and sufficient upper-limb function to hold it safely. A legally authorized representative provided written consent, and resident assent was sought. Exclusions included acute instability; severe sensory, physical, or communication limitations; urgent restraint need; or a major psychiatric disorder unrelated to dementia. Baseline data collection preceded allocation disclosure whenever feasible.

Baseline data included age, sex, education, marital and parental status, dementia type and severity, time since diagnosis, nursing-home length of stay, comorbidity count, social interaction, participation in other therapeutic activities, prior caregiving roles, and prior experience with dolls or pets. Mini-Mental State Examination scores were recorded when available; the instrument provides a brief cognitive descriptor but was not a study outcome (20).

### Randomization and masking

An independent researcher generated the cluster allocation after the baseline assessment using a covariate-constrained process that accounted for facility size, dementia care intensity, staff-to-resident ratio, and aggregate baseline NPI-12. Because the candidate allocation set and balance threshold were unavailable, the randomization sensitivity analysis enumerated all 20 possible allocations of 3 of 6 homes to intervention and was interpreted as unrestricted rather than as a reconstruction of the constrained procedure.

Residents and intervention staff could not be blinded. Outcome assessors were not involved in intervention delivery and were instructed to avoid discussion of assignment. Assessment records included 13 flags indicating assessor awareness, whereas the protocol-deviation log contained 6 unblinding entries. Because the two sources could not be reconciled, they were reported separately; assessment-level flags were used to describe sensitivity. The analysis plan specified that group labels should remain masked until the primary analysis specification was finalized.

### Intervention and comparator

Each intervention participant was offered an individual lifelike doll as an optional comfort and activity object. Staff avoided language requiring the resident or family to accept that it was a real infant. Experienced nurses or nursing assistants completed 8-h training in person-centered care, structured activities, communication, assent and dissent, distress and over-attachment, infection control, and adverse-event procedures, followed by competency assessment.

The introductory phase comprised eight planned 20–30-min sessions on approximately days 1, 3, 5, 7, 9, 11, 13, and 15. Activities included introduction and naming, holding and comforting, reminiscence-oriented conversation, dressing or grooming, storytelling, music, nurturing tasks when welcomed, and planning for continued access. Sessions were individualized to address attention, fatigue, preferences, and emotional responses. After the introductory phase, the same doll remained available during routine care through week 12. Every session was documented for attendance, duration, format, assent, physical and verbal interactions, caregiving behaviors, emotional responses, refusals, distress, adaptations, and fidelity. Independent observers assessed a sample of sessions using an overall fidelity score; item-level observer agreement and Cohen’s kappa were therefore not estimable.

Residents showing refusal or distress had the doll removed or set aside, received reassurance, and were not pressed to continue. Reintroduction was permitted only when the resident was calm and willing. Persistent refusal, distress, possessiveness, interpersonal conflict, or interference with eating, sleep, hygiene, or clinical care triggered individualized review and possible discontinuation of exposure while retaining the resident in the intention-to-treat population. Control homes continued routine nursing, medical, social, and recreational care but did not receive study dolls, training, or the structured doll-therapy protocol. The control condition did not match the eight 20–30-min sessions in terms of staff contact, structured interaction, or activity time. Other clinically indicated non-pharmacological activities and medication reviews were not restricted in either arm and were documented as co-interventions when available. Medication-review schedules were pre-existing facility practices rather than components of the doll-therapy care package; they were neither standardized nor blinded by the study.

### Outcomes

The registered primary outcomes were class-specific mean daily administered doses of antipsychotics, anxiolytics, and antidepressants at baseline, 6 weeks, and 12 weeks. For each time point, scheduled and as-needed administrations during the preceding 7 days were averaged. Antipsychotics were expressed as chlorpromazine daily equivalents. Benzodiazepines and Z-drugs were expressed as diazepam equivalents. Other anxiolytics and antidepressants were quantified using the World Health Organization-defined daily dose framework (21, 22). A dose of zero was assigned only when the medication record was available, and the resident did not receive the class. Unavailable records remained missing. Binary exposure was derived as an available record with a standardized dose greater than zero. Initiation, continuation, and discontinuation statuses were summarized descriptively. For psychotropic polypharmacy, benzodiazepine/Z-drug and other anxiolytic records were combined into a single anxiolytic class; cognitive enhancers were excluded from the psychotropic class count.

The main proximal clinical outcome was NPI-12 total score, calculated as the sum of the 12 domains, with higher scores indicating greater neuropsychiatric symptom burden (3). Activities of daily living were measured using the modified Barthel Index, which ranges from 0 to 100, with higher scores indicating greater independence (23, 24). Cognitive-enhancer exposure was standardized to donepezil daily equivalents (25). Traditional Chinese medicine (TCM) use was summarized as TCM total score = formula-complexity points + daily-dose points + frequency points + duration points. Formula complexity scored 1 for 1–5 herbs, 2 for 6–10, 3 for 11–15, and 4 for >15; daily dose scored 1 for ≤10 g/day, 2 for 11–20 g/day, and 3 for >20 g/day; frequency scored 1 for once daily, 2 for twice daily, and 3 for three or more times daily; and duration scored 1 for <1 month, 2 for 1–3 months, and 3 for >3 months. Non-use scored 0; user totals could range from 4 to 13. Full operationalization is provided in Supplementary Table S9. Because this protocol-defined study-specific score has not been externally validated, it was treated as a contextual descriptor rather than an inferential clinical endpoint.

Feasibility and implementation outcomes included numbers screened and enrolled, 12-week retention, session attendance, the proportion attending at least six of eight introductory sessions, staff authorization, session duration, intervention fidelity, independent observation coverage, weekly engagement-log availability, spontaneous use, refusal and distress episodes, co-interventions, protocol deviations, and adverse events. Adverse events were categorized by severity, seriousness, and relationship to doll therapy.

### Qualitative component

After the intervention period, 24 participants from the 3 intervention homes (15 staff members and 9 family caregivers; eight participants per home) were selected using purposive maximum-variation sampling. The staff sample comprised six registered nurses, six certified nursing assistants, and three activity coordinators, and the participant register included favorable (*n* = 12), mixed/neutral (*n* = 7), and unfavorable (*n* = 5) experiences. All interviews were conducted individually in Mandarin Chinese, audio-recorded with permission, transcribed verbatim, anonymized, and accompanied by an interview-level analytic memo summary. Sampling adequacy was guided by information power rather than a fixed saturation count (26). Analysis followed reflexive thematic analysis (27). The final framework comprised 9 codes and 9 themes applied to 111 transcript-linked excerpts and explicitly represented favorable, mixed/neutral, and unfavorable material, including neutral or absent responses, refusal or distress, implementation workload, and alternative explanations. Each coded excerpt was matched to its source transcript; the nine English quotations reported in the study were source-checked against the corresponding Mandarin text and transcript context. Coder-specific profiles, positionality statements, and formal consensus procedures were not documented prospectively; accordingly, no inter-coder agreement statistic is reported. Reporting was checked against COREQ (28). Aggregated participant distribution, theme coverage, selected de-identified quotations, and qualitative-methods reporting are provided in Supplementary Tables S5–S7 and S10.

### Statistical analysis

All analyses were conducted in R 4.5.1. NPI-12, Barthel, medication dose, traditional Chinese medicine, fidelity, attendance, and per-protocol variables were calculated according to the definitions above. Baseline characteristics were summarized at participant and cluster levels. Continuous variables are presented as mean and standard deviation or median and interquartile range, and categorical variables as count and percentage. Standardized mean differences, rather than baseline significance tests, describe participant-level imbalance.

The primary analysis followed the intention-to-treat principle. For continuous outcomes, marginal Gaussian identity-link models included fixed effects for group, time, and group-by-time interaction and treated nursing home as the top-level cluster with an exchangeable working correlation. Group-by-time coefficients estimate the intervention-minus-control difference in change from baseline at 6 and 12 weeks. The Mancl–DeRouen bias-corrected sandwich estimator was used for the principal variance estimate (29), with 95% confidence intervals and exploratory *p*-values based on a t-distribution with four cluster degrees of freedom. Kauermann–Carroll variance estimates were calculated as part of a sensitivity analysis, and modified GEE estimators were implemented using geesmv (30, 31). A fully iterated delete-one-cluster jackknife produced non-positive variance components with only six clusters and was therefore not used for inference (32). Binary medication exposure was analyzed with log-link Poisson GEE to estimate risk ratios. Because medication-dose distributions were sparse and zero-inflated, exposed counts, zero percentages, medians, and interquartile ranges accompany mean-dose models.

Cluster-level robustness analyses calculated each home’s mean within-participant change among residents with paired baseline and follow-up values and gave each home equal weight. Exact two-sided permutation *p*-values were calculated over all 20 unrestricted 3:3 allocations. Sensitivity analyses for NPI-12 and Barthel used 50 multiple imputations by chained equations under a missing-at-random assumption and stabilized inverse probability of attrition weights based on group, baseline outcome, age, and dementia severity; weights were truncated at the first and 99th percentiles. A *post hoc* descriptive analysis stratified participants by baseline NPI-12 burden using pragmatic score bands: lower (0–20), intermediate (21–40), and higher (41–144). These are not validated clinical severity thresholds. Paired mean change was summarized by arm within each band; subgroup *p*-values were not calculated because only six homes were randomized and *post hoc* categorization increases regression-to-the-mean and multiplicity concerns. Per-protocol descriptive analyses used the prespecified threshold of at least six introductory sessions. No adjustment for multiple testing was made because the trial was exploratory. Missingness, unrestricted permutation, MICE-MAR, attrition-weighted, and NPI-stratified results are reported in Supplementary Tables S1–S4 and S8.

Quantitative and qualitative results were integrated in a joint display. Relationships were labeled convergence when the components supported a similar interpretation, complementarity when interviews explained context or implementation behind a quantitative result, and dissonance when accounts emphasized heterogeneity or an interpretation not evident in group averages.

### Ethics

The study was approved by the Institutional Review Board of the School of Medicine, Sias University (LL20240509) and registered prospectively at ClinicalTrials.gov (NCT06506487; July 16, 2024). Legally authorized representatives provided written informed consent for residents, and ongoing verbal and non-verbal assent or dissent was respected. Interview participants provided separate written consent. Trial reporting was checked against CONSORT 2025 (33), the cluster extension (34), and the pilot and feasibility extension (35).

## Results

### Recruitment, retention, and baseline characteristics

**Figure 1:**
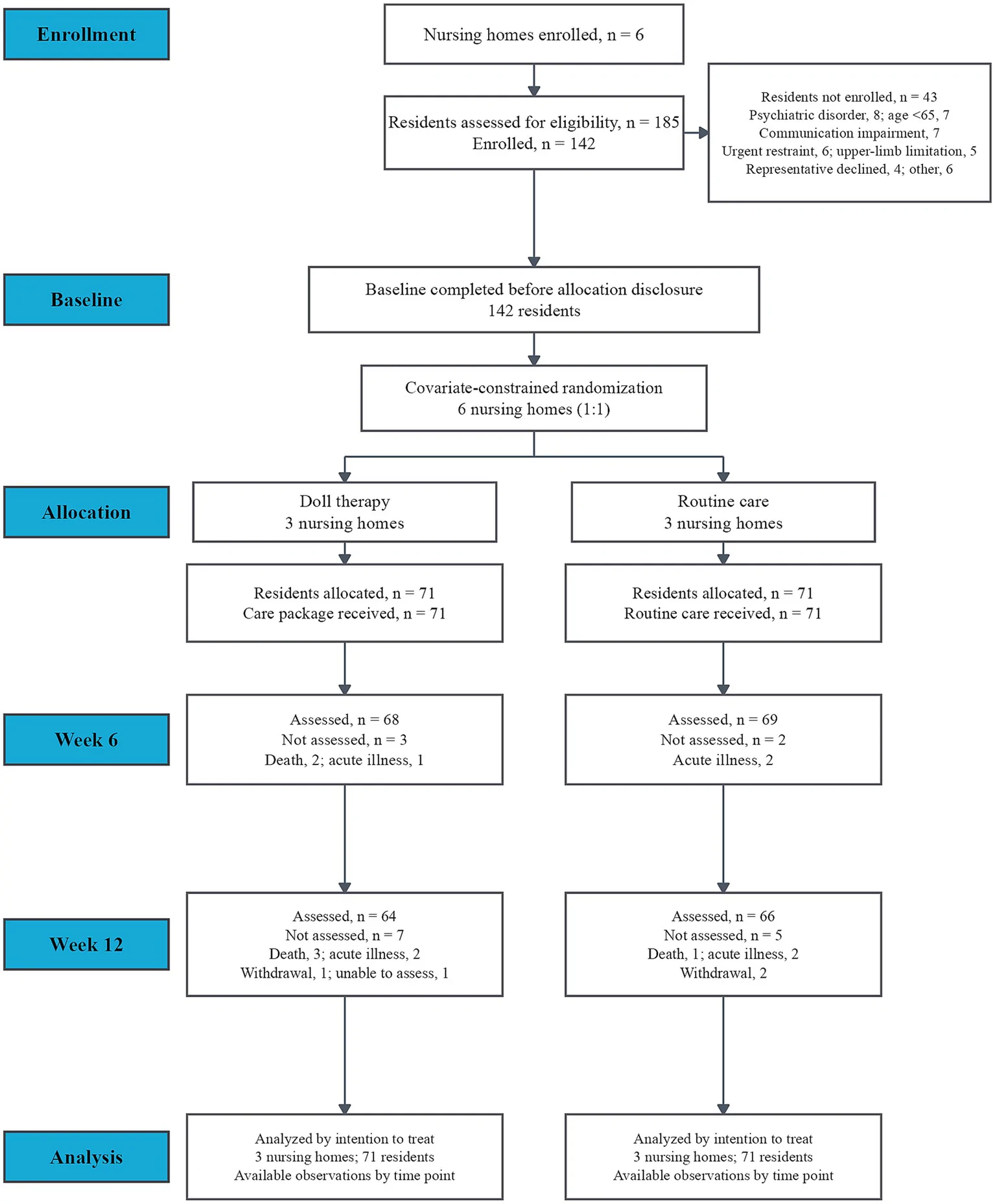
Participant and cluster flow. The diagram distinguishes nursing-home enrollment and allocation from resident screening, follow-up, and analysis. The ‘other’ non-enrollment category comprised no available representative (*n* = 3), acute instability (*n* = 2), and visual impairment (*n* = 1). All 6 randomized homes and 142 residents remained in the intention-to-treat population; outcome models used available observations, and unavailable medication records were not coded as zero.

Six nursing homes were randomized, with 23–24 residents enrolled per home. Of 185 residents screened, 142 were enrolled (76.8%; 95% CI, 70.0–82.6%). Forty-three were not enrolled because of eligibility, safety, communication, representative, or consent criteria detailed in Figure 1. Seventy-one residents entered each arm.

The mean age was 83.4 years (SD, 6.9), and 93 participants (65.5%) were women. Baseline NPI-12 was 31.4 (SD, 12.9) in the intervention arm and 32.6 (SD, 11.8) in the control arm. Baseline Barthel scores were 40.2 (SD, 22.1) and 43.7 (SD, 20.0), respectively. Participant-level imbalances were most apparent for dementia type and severity: severe dementia was more common in the intervention arm (40.8% vs. 26.8%; standardized mean difference, 0.30), while vascular dementia was less common (18.3% vs. 32.4%; standardized mean difference, −0.33). Cluster-level baseline mean NPI-12 ranged from 29.83 to 34.43. Table 1 presents participant and home characteristics.

**Table 1:** Nursing home and participant baseline characteristics.

Panel A. Nursing-home characteristics									
Home	Arm	Enrolled, *n*	Beds, *n*	Residents with dementia, *n*	Dementia-care intensity	Direct-care staff, *n*	Staff-to-resident ratio	Medication-review frequency	Baseline NPI-12, mean
NH01	Intervention	24	168	78	46.4%	56	0.718	Monthly	30.62
NH02	Control	24	152	70	46.1%	51	0.729	Quarterly	33.54
NH03	Control	23	196	91	46.4%	62	0.681	As needed	34.43
NH04	Intervention	24	180	83	46.1%	59	0.711	Monthly	30.54
NH05	Control	24	144	64	44.4%	47	0.734	Quarterly	29.83
NH06	Intervention	23	174	80	46.0%	57	0.713	Monthly	33.17
Dementia-care intensity is the number of residents with dementia divided by the number of licensed beds. The staff-to-resident ratio is the number of direct-care staff divided by the number of residents with dementia.									

Panel B. Participant baseline characteristics				
Characteristic	Overall (*n* = 142)	Intervention (*n* = 71)	Control (*n* = 71)	SMD
Age, years	83.4 (6.9)	83.4 (7.2)	83.3 (6.7)	0.02
Sex: F	93 (65.5%)	47 (66.2%)	46 (64.8%)	0.03
Education: None	27 (19.0%)	14 (19.7%)	13 (18.3%)	0.04
Education: Primary	58 (40.8%)	27 (38.0%)	31 (43.7%)	−0.11
Education: Middle	37 (26.1%)	20 (28.2%)	17 (23.9%)	0.1
Education: High School+	20 (14.1%)	10 (14.1%)	10 (14.1%)	0
Dementia type: Alzheimer’s	77 (54.2%)	43 (60.6%)	34 (47.9%)	0.26
Dementia type: Vascular	36 (25.4%)	13 (18.3%)	23 (32.4%)	−0.33
Dementia type: Mixed	20 (14.1%)	11 (15.5%)	9 (12.7%)	0.08
Dementia type: Other/unspecified	9 (6.3%)	4 (5.6%)	5 (7.0%)	−0.06
Dementia severity: Mild	24 (16.9%)	11 (15.5%)	13 (18.3%)	−0.08
Dementia severity: Moderate	70 (49.3%)	31 (43.7%)	39 (54.9%)	−0.23
Dementia severity: Severe	48 (33.8%)	29 (40.8%)	19 (26.8%)	0.3
Years since dementia diagnosis	6.0 (3.0)	6.3 (3.1)	5.7 (2.9)	0.19
Nursing-home stay, months	31.4 (21.2)	30.9 (20.8)	31.9 (21.7)	−0.05
Comorbidity count	3.2 (1.2)	3.3 (1.1)	3.1 (1.3)	0.17
Other therapeutic activities per week	2.5 (1.2)	2.4 (1.1)	2.5 (1.3)	−0.08
Prior caregiving role: Yes	90 (63.4%)	47 (66.2%)	43 (60.6%)	0.12
Prior doll or pet experience: Yes	68 (47.9%)	35 (49.3%)	33 (46.5%)	0.06
Baseline NPI-12	32.0 (12.4)	31.4 (12.9)	32.6 (11.8)	−0.09
Baseline Barthel Index	41.9 (21.1)	40.2 (22.1)	43.7 (20.0)	−0.16

Values are mean (SD) or *n* (%). All observed education and dementia-type categories are shown. SMD, standardized mean difference; NPI-12, 12-domain Neuropsychiatric Inventory.

At week 6, assessments were completed for 68 intervention and 69 control residents. At week 12, 64 intervention and 66 control residents completed follow-up, yielding a retention rate of 91.5% (130/142; 95% CI, 85.7–95.6%). Non-completion reflected death, hospitalization, or acute deterioration, representative withdrawal, or inability to assess. Medication records were unavailable for nine participant-time points and remained missing. Arm-by-time missingness is shown in Supplementary Table S1.

### Feasibility, exposure, fidelity, and safety

All 18 trained intervention deliverers were authorized. Intervention participants attended 508 of 568 planned sessions (89.4%; 95% CI, 86.6–91.8%). Sixty-eight of 71 residents (95.8%; 95% CI, 88.1–99.1%) attended at least six sessions. The median number attended was seven; 29 residents attended all eight, 28 attended seven, 11 attended six, two attended five, and one attended four. Reasons for 60 missed sessions were fatigue (*n* = 21), resident unavailability (*n* = 15), acute illness (*n* = 14), and staffing constraints (*n* = 10). The mean attended-session duration was 23.9 min, and mean fidelity was 94.1%. Independent observers assessed 153 attended sessions (30.1%), exceeding 20% in each intervention home. Weekly engagement logs were available for 690 of 710 expected participant-weeks (97.2%). Routine logs recorded 44 refusal episodes, 42 distress episodes, and 26 possessiveness or interpersonal-conflict episodes. These counts represent events, not unique residents.

Seventeen adverse events were recorded: 12 in the intervention arm and five in the control arm. Six intervention events were judged possibly or probably related to doll therapy; three were mild and possibly related, two were moderate and possibly related, and one was moderate and probably related. No serious event was related to the intervention. The control arm had two serious events, both judged unrelated, and three mild unrelated events. Sixteen protocol deviations and 27 co-interventions were documented. Medication reviews occurred more often among recorded intervention co-interventions, but these events were not randomized and were interpreted descriptively (Table 2).

**Table 2:** Feasibility, implementation, and safety outcomes.

Metric	Numerator	Denominator	Estimate/value	95% CI or note
Recruitment among screened residents	142	185	76.8%	70.0–82.6%
Completed 12-week follow-up, overall	130	142	91.5%	85.7–95.6%
Completed 12-week follow-up, intervention	64	71	90.1%	80.7–95.9%
Completed 12-week follow-up, control	66	71	93.0%	84.3–97.7%
Intervention participants attending ≥6/8 sessions	68	71	95.8%	88.1–99.1%
Planned introductory sessions attended	508	568	89.4%	86.6–91.8%
Attended sessions independently observed	153	508	30.1%	26.2–34.3%
Routine engagement weekly logs available	690	710	97.2%	95.7–98.3%
Authorized intervention deliverers			18	Descriptive
Mean attended-session duration, min			23.9	Descriptive
Mean attended-session fidelity			94.1%	Descriptive
Adverse events, intervention			12	Descriptive
Adverse events, control			5	Descriptive
Serious adverse events, intervention			0	Descriptive
Serious adverse events, control			2	Descriptive
Possible/probable doll-related adverse events			6	Descriptive
Protocol deviations			16	Descriptive
Co-interventions			27	Descriptive

### Main proximal and secondary clinical outcomes

**Figure 2:**
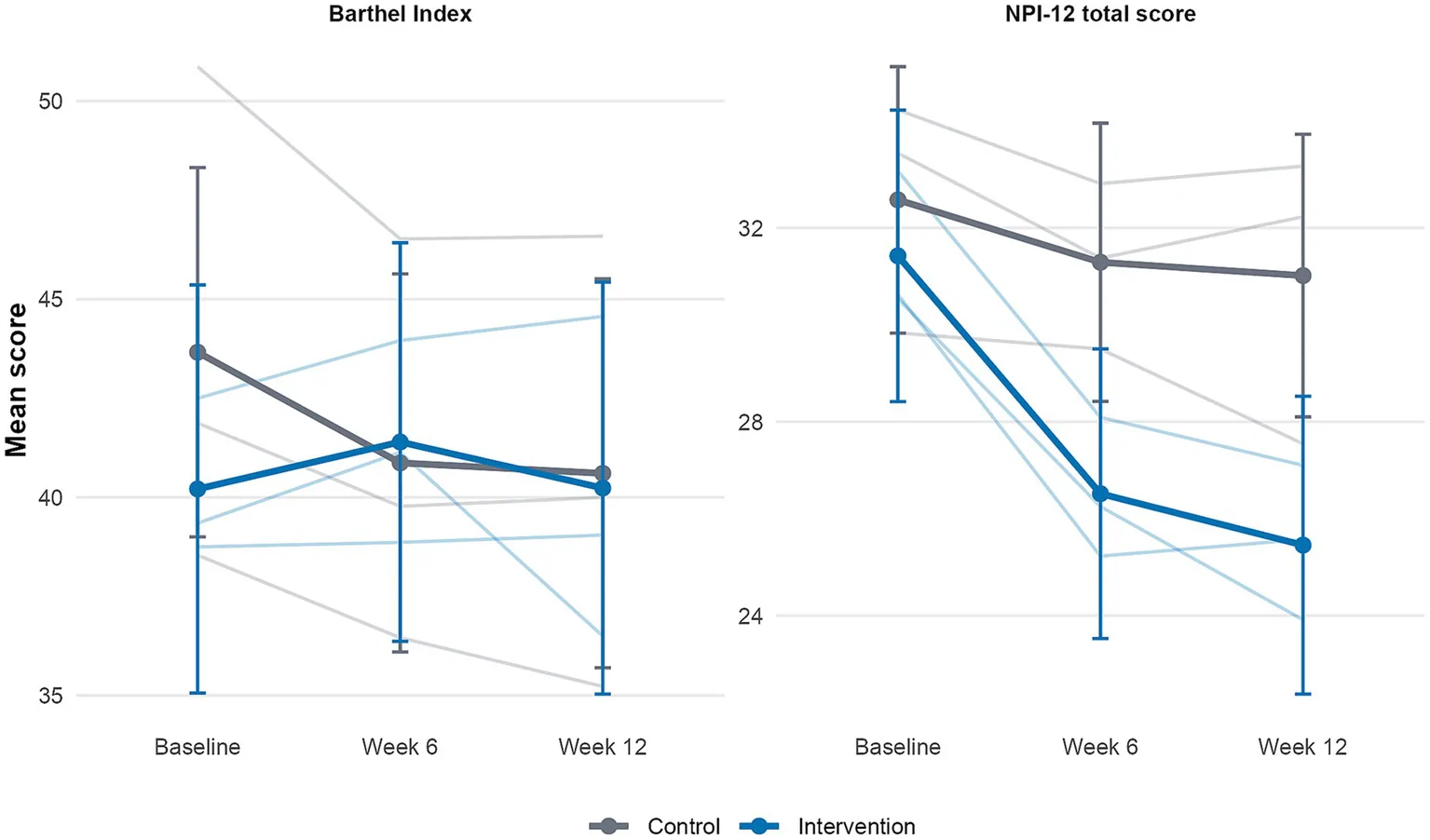
Clinical outcome trajectories. Participant-level arm means and 95% confidence intervals for NPI-12 and Barthel Index at baseline, 6 weeks, and 12 weeks. Faint lines show nursing-home means. Lower NPI-12 scores indicate fewer neuropsychiatric symptoms; higher Barthel scores indicate greater independence.

Mean NPI-12 declined in both groups, with a larger change in the intervention arm (Figure 2). In Figure 2, thick lines denote participant-level arm means with 95% confidence intervals, whereas faint lines show nursing-home means. At week 6, mean NPI-12 was 26.5 (SD, 12.6) in the intervention arm and 31.3 (SD, 12.2) in the control arm. The model-estimated intervention-control difference in change was −3.61 points (95% CI, −5.86 to −1.36; exploratory *p* = 0.011). At week 12, means were 25.5 (SD, 12.5) and 31.0 (SD, 12.1), and the difference in change was −4.40 points (95% CI, −6.50 to −2.30; exploratory *p* = 0.004). Equal-weighted cluster change estimates were −3.53 at week 6 and −4.98 at week 12; each had an unrestricted exact permutation *p*-value of 0.10, the smallest attainable two-sided value for the observed ordering over 20 allocations. Multiple-imputation and attrition-weighted estimates were directionally consistent (Supplementary Tables S2–S4).

In the *post hoc* analysis stratified by baseline NPI-12 burden, counts were 13 interventions and 11 control residents for the lower band, 38 vs. 40 for the intermediate band, and 20 vs. 20 for the higher band. Among residents with paired measurements, intervention-minus-control differences in mean change were −3.88, −3.45, and −3.39 points at week 6 and −2.30, −5.80, and −4.78 points at week 12 for the lower, intermediate, and higher bands, respectively. The descriptive direction was similar across bands, but these pragmatic categories are not validated severity thresholds, and the analysis was not used for subgroup inference (Supplementary Table S8).

**Figure 3:**
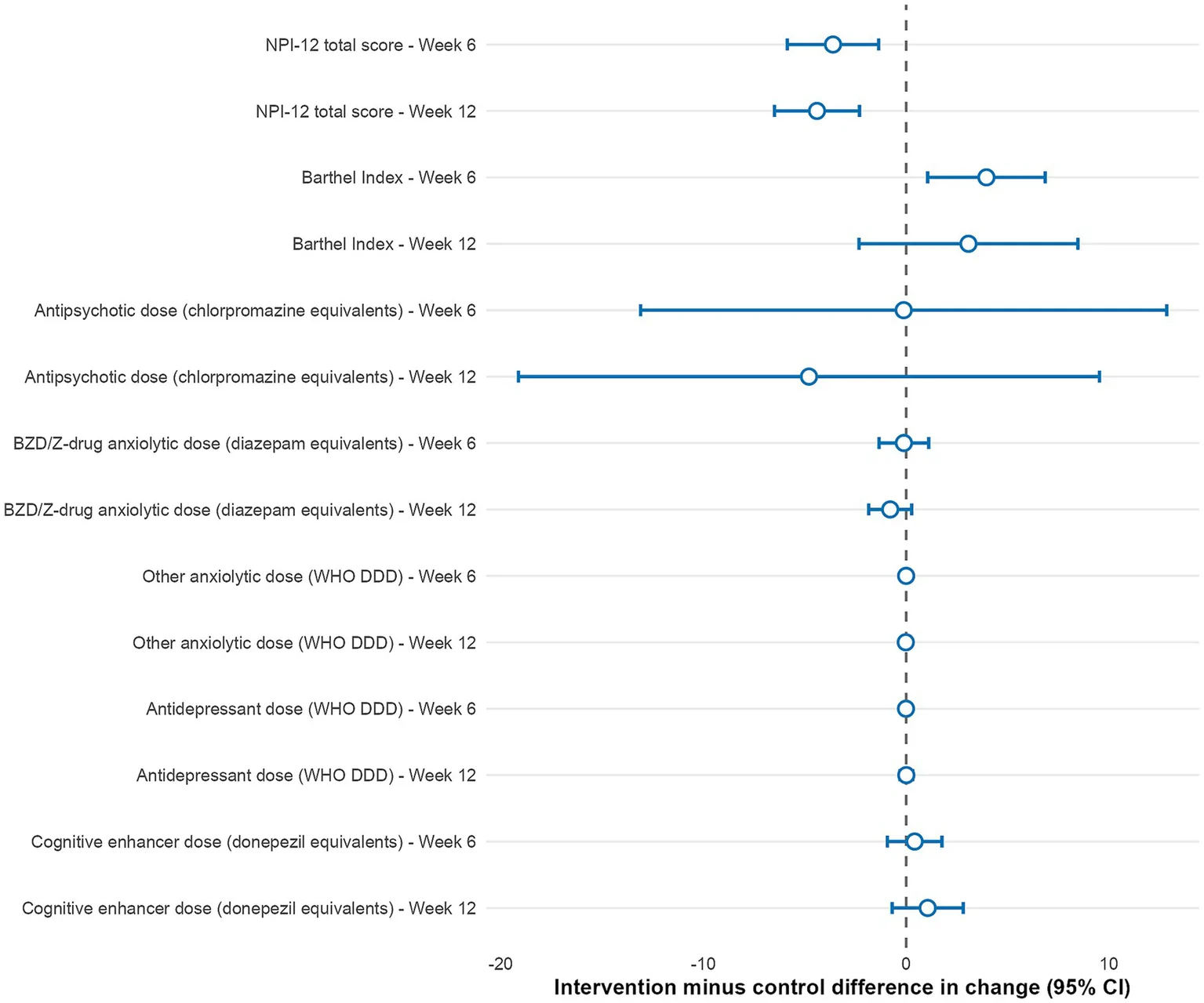
Exploratory marginal treatment-effect estimates. Intervention-minus-control differences in change from baseline at 6 and 12 weeks with 95% confidence intervals calculated using the Mancl–DeRouen variance correction and a *t*-distribution with four cluster degrees of freedom. Estimates are exploratory because six homes were randomized and outcomes use different units.

Mean Barthel scores were 41.4 (SD, 21.2) in the intervention arm and 40.9 (SD, 20.2) in the control arm at week 6, producing a difference in change of 3.96 points (95% CI, 1.06 to 6.86; exploratory *p* = 0.019). At week 12, means were 40.2 (SD, 21.2) and 40.6 (SD, 20.3); the difference in change was 3.07 points (95% CI, −2.33 to 8.47; exploratory *p* = 0.189). Cluster-level and imputed analyses suggested a positive direction, but the attrition-weighted week-12 interval included no difference. Cognitive-enhancer dose effects also remained imprecise: the difference in change was 0.42 mg/day at week 6 (95% CI, –0.93 to 1.76) and 1.06 mg/day at week 12 (95% CI, –0.69 to 2.81). Table 3 summarizes these clinical estimates. Figure 3 plots the exploratory intervention-minus-control differences in change and their small-sample-corrected 95% confidence intervals across outcome scales.

**Table 3:** Clinical outcomes and intervention-control differences in change.

Outcome	Time	Intervention mean (SD)	Control mean (SD)	Difference in change	95% CI	Exploratory *p*
NPI-12 total score	Week 6	26.5 (12.6)	31.3 (12.2)	−3.61	−5.86 to −1.36	0.011
NPI-12 total score	Week 12	25.5 (12.5)	31.0 (12.1)	−4.40	−6.50 to −2.30	0.004
Barthel Index	Week 6	41.4 (21.2)	40.9 (20.2)	3.96	1.06 to 6.86	0.019
Barthel Index	Week 12	40.2 (21.2)	40.6 (20.3)	3.07	−2.33 to 8.47	0.189
Cognitive-enhancer dose, donepezil-equivalent mg/day	Week 6	4.9 (4.1)	3.9 (4.0)	0.42	−0.93 to 1.76	0.438
Cognitive-enhancer dose, donepezil-equivalent mg/day	Week 12	5.3 (4.3)	3.7 (4.1)	1.06	−0.69 to 2.81	0.168

Differences are intervention-minus-control changes from baseline. Confidence intervals use the Mancl–DeRouen variance correction and a t-distribution with four cluster degrees of freedom. *p*-values are exploratory.

### Registered medication outcomes and contextual treatment

For antipsychotics, the difference in standardized dose change was −0.12 chlorpromazine-equivalent mg/day at week 6 (95% CI, −13.10 to 12.85) and −4.79 mg/day at week 12 (95% CI, −19.12 to 9.53). The antipsychotic exposure log-Poisson GEE did not converge, so no exposure risk ratio or model-based *p*-value was reported; exposed counts and zero proportions are presented descriptively.

For benzodiazepine/Z-drug anxiolytics, the difference in diazepam-equivalent change was −0.11 mg/day at week 6 (95% CI, −1.34 to 1.11) and −0.79 mg/day at week 12 (95% CI, −1.85 to 0.27). Other anxiolytic DDD differences were −0.002 (95% CI, −0.053 to 0.048) and −0.020 (95% CI, −0.112 to 0.072). Antidepressant DDD differences were −0.010 (95% CI, −0.144 to 0.124) and 0.006 (95% CI, −0.311 to 0.323). Unrestricted cluster permutation results indicated substantial uncertainty in medication outcomes. Usual medication-review frequency was monthly in all three intervention homes, quarterly in two control homes, and as needed in one control home (Table 1). This cluster-level imbalance predated allocation, was not part of the doll-therapy protocol, and was neither randomized nor blinded; with only six homes, it was inseparable from study arm. At week 12, no psychotropic exposure was recorded for 32.8% of intervention residents with complete medication data and 19.1% of control residents. Because class exposure and review opportunities differed at baseline and records were missing for some residents, this contrast was treated as descriptive and cannot be attributed specifically to doll therapy. Table 4 presents doses, exposure transitions, and polypharmacy patterns. The study-specific traditional Chinese medicine score was summarized as contextual treatment and was not used for a clinical-effect claim.

**Table 4:** Registered medication outcomes.

Panel A. Dose distributions and model estimates					
Medication class and unit	Time	Intervention: available/Exposed n; mean; median [IQR]; zero %	Control: available/Exposed *n*; mean; median [IQR]; zero %	Difference in change (95% CI)	Exposure RR (95% CI)
Antipsychotic, chlorpromazine-equivalent mg/day	Baseline	71/27; 34.15; 0.00 [0.00–75.00]; 62.0%	71/28; 33.79; 0.00 [0.00–71.00]; 60.6%	Not applicable	Not applicable
Antipsychotic, chlorpromazine-equivalent mg/day	Week 6	69/21; 30.85; 0.00 [0.00–50.00]; 69.6%	71/28; 30.59; 0.00 [0.00–50.00]; 60.6%	−0.12 (−13.10 to 12.85)	Not estimated
Antipsychotic, chlorpromazine-equivalent mg/day	Week 12	67/23; 32.08; 0.00 [0.00–75.00]; 65.7%	68/27; 36.55; 0.00 [0.00–76.72]; 60.3%	−4.79 (−19.12 to 9.53)	Not estimated
Benzodiazepine/Z-drug anxiolytic, diazepam-equivalent mg/day	Baseline	71/19; 1.39; 0.00 [0.00–1.25]; 73.2%	71/22; 1.20; 0.00 [0.00–1.88]; 69.0%	Not applicable	Not applicable
Benzodiazepine/Z-drug anxiolytic, diazepam-equivalent mg/day	Week 6	69/23; 1.34; 0.00 [0.00–2.50]; 66.7%	71/21; 1.26; 0.00 [0.00–1.88]; 70.4%	−0.11 (−1.34 to 1.11)	1.31 (0.97–1.77)
Benzodiazepine/Z-drug anxiolytic, diazepam-equivalent mg/day	Week 12	67/16; 0.90; 0.00 [0.00–0.00]; 76.1%	68/23; 1.48; 0.00 [0.00–2.74]; 66.2%	−0.79 (−1.85 to 0.27)	0.81 (0.44–1.48)
Other anxiolytic, WHO DDD/day	Baseline	71/8; 0.07; 0.00 [0.00–0.00]; 88.7%	71/5; 0.05; 0.00 [0.00–0.00]; 93.0%	Not applicable	Not applicable
Other anxiolytic, WHO DDD/day	Week 6	69/7; 0.07; 0.00 [0.00–0.00]; 89.9%	71/5; 0.05; 0.00 [0.00–0.00]; 93.0%	−0.00 (−0.05 to 0.05)	0.91 (0.69–1.20)
Other anxiolytic, WHO DDD/day	Week 12	67/7; 0.06; 0.00 [0.00–0.00]; 89.6%	68/5; 0.06; 0.00 [0.00–0.00]; 92.6%	−0.02 (−0.11 to 0.07)	0.89 (0.61–1.31)
Antidepressant, WHO DDD/day	Baseline	71/24; 0.29; 0.00 [0.00–0.50]; 66.2%	71/26; 0.32; 0.00 [0.00–0.50]; 63.4%	Not applicable	Not applicable
Antidepressant, WHO DDD/day	Week 6	69/22; 0.28; 0.00 [0.00–0.50]; 68.1%	71/28; 0.32; 0.00 [0.00–0.50]; 60.6%	−0.01 (−0.14 to 0.12)	0.88 (0.75–1.02)
Antidepressant, WHO DDD/day	Week 12	67/20; 0.28; 0.00 [0.00–0.50]; 70.1%	68/25; 0.32; 0.00 [0.00–0.50]; 63.2%	0.01 (−0.31 to 0.32)	0.88 (0.60–1.29)
Zero percentages and dose distributions are based on available medication records. A zero denotes an available record with no administered medication in that class. The antipsychotic exposure model did not converge and was not estimated. DDD, defined daily dose; IQR, interquartile range; RR, risk ratio.					

Panel B. Medication-class exposure transitions from baseline to week 12						
Medication class	Arm	Available at both times, *n*	Continued	Discontinued	Initiated	No exposure at either time
Antipsychotic, chlorpromazine-equivalent mg/day	Intervention	67	21 (31.3%)	4 (6.0%)	2 (3.0%)	40 (59.7%)
Antipsychotic, chlorpromazine-equivalent mg/day	Control	68	25 (36.8%)	1 (1.5%)	2 (2.9%)	40 (58.8%)
Benzodiazepine/Z-drug anxiolytic, diazepam-equivalent mg/day	Intervention	67	13 (19.4%)	6 (9.0%)	3 (4.5%)	45 (67.2%)
Benzodiazepine/Z-drug anxiolytic, diazepam-equivalent mg/day	Control	68	21 (30.9%)	1 (1.5%)	2 (2.9%)	44 (64.7%)
Other anxiolytic, WHO DDD/day	Intervention	67	7 (10.4%)	0 (0.0%)	0 (0.0%)	60 (89.6%)
Other anxiolytic, WHO DDD/day	Control	68	5 (7.4%)	0 (0.0%)	0 (0.0%)	63 (92.6%)
Antidepressant, WHO DDD/day	Intervention	67	20 (29.9%)	2 (3.0%)	0 (0.0%)	45 (67.2%)
Antidepressant, WHO DDD/day	Control	68	22 (32.4%)	2 (2.9%)	3 (4.4%)	41 (60.3%)
Percentages use residents with an available class record at both baseline and week 12 as the denominator						

Panel C. Psychotropic polypharmacy						
Arm	Time	Available, *n*	No exposure	Monotherapy	Two classes	Three classes
Intervention	Baseline	71	19 (26.8%)	29 (40.8%)	22 (31.0%)	1 (1.4%)
Intervention	Week 6	69	22 (31.9%)	25 (36.2%)	21 (30.4%)	1 (1.4%)
Intervention	Week 12	67	22 (32.8%)	27 (40.3%)	16 (23.9%)	2 (3.0%)
Control	Baseline	71	14 (19.7%)	38 (53.5%)	15 (21.1%)	4 (5.6%)
Control	Week 6	71	14 (19.7%)	37 (52.1%)	16 (22.5%)	4 (5.6%)
Control	Week 12	68	13 (19.1%)	36 (52.9%)	14 (20.6%)	5 (7.4%)

The psychotropic class count combines benzodiazepine/Z-drug and other anxiolytic records into one anxiolytic class; cognitive enhancers are excluded. Percentages use complete class records.

### Qualitative findings and mixed-methods integration

Twenty-four people participated in qualitative interviews: 15 staff members and 9 family caregivers, with 5 staff and 3 family participants from each intervention home. Staff interviews averaged 44.5 min and family interviews 40.4 min. All 111 coded excerpts were matched to their source transcript. Nine interrelated themes were identified (Table 5; Supplementary Tables S5–S7).

**Table 5:** Qualitative themes and representative quotations.

Theme	Participants	Interpretation	Representative quotation
Acceptability and first impressions	13	Acceptability depended on an optional, non-deceptive introduction.	In routine care at NH01, I initially found it somewhat novel and worried that residents might not accept it. After staff explained that it was an optional comfort and activity object, I thought it was worth trying.
Alternative explanations	12	Staff attention, environment, health, and medication change complicated attribution.	From what we observed over these months, the improvement may also have come from staff spending more than 10 extra minutes with the resident or from a quieter environment that day; it was not necessarily due entirely to the doll.
Cultural fit and family communication	9	Language and family explanation shaped cultural fit.	In routine care at NH01, describing it as a comfort and activity object rather than pretending it was a real infant was more acceptable to families. [Pause] It depends on the individual.
Dignity, autonomy and ethics	15	Dignity was linked to privacy and respect for real-time refusal.	From what we observed over these months, I think dignity depends not on the object itself, but on how staff introduces it, whether refusal is respected, and whether privacy is protected.
Implementation workload	12	Cleaning, storage, documentation, and ownership affected sustainability.	From my perspective as a certified nursing assistant, without a designated person in charge, cleaning, storage, and documentation for the dolls may all be neglected. I do not think the current process is sufficiently sustainable.
Neutral or absent response	9	No response was valid, prompting the use of alternative activities.	From my perspective as a certified nursing assistant, some residents merely glanced at the doll and did not reach for it. Staff documented this and then switched to music or conversation rather than treating the absence of a response as a failure.
Observed engagement and affect	15	Some residents showed nurturing interaction and calmer affect.	From my perspective as a certified nursing assistant, she once held the doll, spoke softly, and adjusted its clothes on her own. Her expression was noticeably softer than before the activity, and this lasted for more than 10 min.
Refusal, distress and stopping rules	16	Prompt stopping after verbal or non-verbal refusal was essential.	From what I observed during implementation over the past few months, I saw a resident push the doll away, but staff asked her twice more. Although no one forced her, she became visibly more agitated. I think staff should stop more decisively.
Training and fidelity	10	Role-play supported communication skills; practice coaching remained important.	From my perspective as a registered nurse, role-play was the most helpful part. It allowed us to practice avoiding coaxing or commanding language and to learn how to recognize non-verbal refusal.

Quotations were translated from Mandarin Chinese and source-checked against the corresponding transcripts; de-identified Mandarin text and translation-verification status are provided in Supplementary Table S7.

First, acceptability was conditional rather than inherent. Cultural fit was linked to non-deceptive language and individualized family communication.

Second, observed engagement and affect varied. Staff described residents holding the doll, speaking softly, adjusting its clothes, or appearing calmer for a brief period. Others looked once without reaching, ignored the doll, or preferred music or conversation. Staff who treated a lack of response as information rather than a failure described switching activities without pressure. These accounts converged with the group-level NPI-12 signal but cautioned against assuming a universal response or attributing functional improvement to doll therapy.

Third, alternative explanations were prominent. Interviewees noted that extra staff time, a quieter environment, changes in physical health, medication review, and ordinary fluctuation could explain apparent improvement. This complemented the quantitative finding that behavioral scores changed while medication-dose effects remained imprecise.

Fourth, training and operational ownership shaped fidelity. Staff requested more on-site demonstration for conflict or over-attachment and warned that cleaning, storage, and documentation could become unsustainable without a designated responsible person. High recorded fidelity, therefore, coexisted with implementation work that would require explicit resourcing in a larger trial.

Finally, dignity, assent, and stopping rules were central to ethical acceptability. Participants stated that dignity depended on how the intervention was introduced, whether privacy was protected, and whether refusal was respected. The joint display (Table 6) summarizes convergence, complementarity, and dissonance across components; the negative case remains visible in Table 5.

**Table 6:** Joint display integrating quantitative and qualitative findings.

Domain	Quantitative finding	Qualitative themes	Integration type	Integrated interpretation
Behavioral symptoms	Week-12 NPI-12 difference in change: −4.40 (95% CI -6.50 to −2.30)	Observed engagement and affect; Alternative explanations	Convergence with cautious attribution	Staff and family observations support a behavioral signal and attribute the change to increased attention and calmer interactions.
Activities of daily living	Week-12 Barthel difference in change 3.07 (95% CI -2.33 to 8.47)	Neutral or absent response; observed engagement and affect	Dissonance and heterogeneity	Functional change was limited, and participants varied in interest, supporting individualized rather than universal use.
Medication stewardship	Week-12 antipsychotic dose difference in change −4.79 (95% CI -19.12 to 9.53)	Alternative explanations; implementation workload	Complementarity	Prescribing remained clinician- and facility-dependent; interviews explained why behavioral change may not translate directly into dose reduction.
Engagement and fidelity	95.8% attended at least six sessions; mean fidelity 94.1%	Training and fidelity; acceptability and first impressions	Convergence	High exposure was feasible when staff were trained, but workload and need for practical coaching remained salient.
Safety and ethics	6 possible/probable doll-related adverse events; no serious doll-related event	Refusal, distress and stopping rules; dignity, autonomy and ethics	Complementarity	Stopping rules and non-deceptive, dignity-preserving delivery were central to acceptability and safe implementation.

Convergence indicates similar interpretations across components; complementarity indicates that qualitative findings explain context or implementation behind a quantitative result; dissonance indicates heterogeneity or a contrasting interpretation.

## Discussion

This exploratory mixed-methods cluster trial found that a structured, staff-mediated doll-therapy care package was feasible to implement in three Chinese nursing homes, with high introductory-session exposure, strong recorded fidelity, and extensive routine engagement documentation. NPI-12 showed a directionally consistent intervention signal at 6 and 12 weeks across participant-level marginal models, cluster summaries, multiple imputation, and attrition weighting. Barthel scores showed an early favorable difference but did not provide a precise sustained signal at 12 weeks. Registered medication-dose outcomes were sparse and imprecise, with all confidence intervals including no difference. No serious adverse event was related to doll therapy, although mild and moderate possibly or probably related events, refusals, distress, and conflict were documented. Interviews clarified that benefit was heterogeneous and that ethical delivery required training, non-deceptive introduction, active monitoring of dissent, family communication, and operational responsibility.

The behavioral finding is consistent with, but does not confirm, the growing doll-therapy literature. The 2024 meta-analysis reported pooled improvements in behavioral and psychological outcomes, particularly agitation, but found no cognitive benefit (12). A randomized study of 52 nursing-home residents also reported reductions in agitation, aggression, dysphoria, wandering, and apathy (36). The present trial extends the literature by using cluster allocation, a 12-domain standardized outcome, repeated follow-up, fidelity data, medication records, safety monitoring, and qualitative interpretation in Chinese residential care. The estimated NPI-12 differences were modest relative to the broad baseline variability, and the confidence intervals reflect only model-based uncertainty under strong assumptions. With three homes per arm, site culture or routine practice could produce a similar pattern. The exact permutation analysis appropriately returned *p* = 0.10 despite narrower model-based intervals, illustrating why the finding should be described as a preliminary behavioral signal rather than proof of efficacy.

One plausible explanation for the NPI-12 pattern is that the care package created repeated opportunities for calm, individualized, emotionally meaningful interaction. Attachment-informed caregiving behaviors, role continuity, sensory contact, and staff attention may each contribute. The routine-care comparator did not provide a time-and-attention-matched activity, so the additional 20–30 min per introductory session could have affected NPI-12 through social contact, individualized reassurance, structured activity, expectations, or a temporarily calmer care context even if the doll itself had no specific effect. Repeated contact may also have changed how staff interacted with residents outside sessions, as well as the behavioral changes they noticed and documented. Because the doll, training, structured interaction, and added staff time were delivered together, the present estimate applies to the care package and cannot isolate a doll-specific mechanism. Interview participants explicitly attributed some observed change to extra time, environmental calm, or the way staff approached the resident. The WHELD cluster trial combined person-centered training, social interaction, activity, and antipsychotic review, improving quality-of-life and agitation outcomes (37). A future attention-control arm that offers equally structured staff interaction without a doll would help separate doll-specific meaning from attention and activity effects.

The absence of a sustained Barthel signal was also coherent with the intervention’s proposed mechanism. Doll therapy primarily targeted affect regulation, distress, and engagement rather than mobility, continence, transfers, or other components of the Barthel Index. The early difference could reflect temporary engagement, ordinary fluctuation, baseline imbalance, missingness, or site effects. Interviewees frequently described brief changes in expression or interaction rather than generalized functional independence. A larger trial should retain activities of daily living as a secondary outcome but consider additional proximal measures such as observed engagement, affect, agitation episodes, quality of interaction, or goal-attainment measures tailored to person-centered care. The lack of a cognitive hypothesis is consistent with meta-analytic evidence indicating no clear doll-therapy effect on cognition (12).

Medication findings require especially cautious interpretation. The observed direction for week-12 benzodiazepine/Z-drug dose favored intervention, but its interval included no difference, and other class-specific estimates were similarly imprecise. All intervention homes had monthly routine medication reviews, whereas control homes used quarterly or as-needed review. This pre-existing site characteristic was completely confounded with arm; prescribers were not directed by the doll-therapy protocol, and their masking to residents’ activities could not be assured. No adjusted analysis can separate doll therapy from review opportunity with three homes per arm. Because class-specific dose outcomes were registered primary outcomes, they are reported here but should be interpreted only as imprecise service-delivery signals; no pharmacological treatment-effect claim is warranted. A medication change requires a prescriber, a review opportunity, clinical confidence that symptoms are stable, and agreement with staff and family. Enhanced psychosocial care has reduced antipsychotic use when staff practice was explicitly targeted (38), and the COSMOS trial demonstrated that a multicomponent medication-review intervention can reduce psychotropic exposure without worsening neuropsychiatric symptoms (39). In contrast, a recent cluster trial targeting antidepressant deprescribing reported an odds ratio of 2.3 but a wide confidence interval that included no difference (40), while a 2026 staff-training trial reported mixed agitation findings alongside favorable psychotropic-use outcomes (41). These comparisons suggest that medication reduction is most plausible when prescribing processes are targeted directly. Doll therapy may contribute behavioral information or a non-pharmacological option, but it should not be expected to change medication use reliably without a structured stewardship pathway.

The qualitative findings also explain why average intervention effects may conceal meaningful heterogeneity. Some residents displayed nurturing behavior or calm engagement, whereas others showed little interest or active refusal. Prior reviews have raised concerns about infantilization, deception, and over-attachment (16, 17). Participants linked acceptability to language, privacy, family understanding, and respect for real-time dissent. An ethical implementation model therefore requires more than proxy consent. In this study, that meant observing facial expressions, body movements, pushing away, turning away, fear, crying, or escalating agitation and responding immediately. Non-response remained a valid outcome, and alternative activities were kept available. A future trial should predefine ethical fidelity indicators alongside procedural fidelity and report the number and management of refusals, distress episodes, and discontinued exposures.

High attendance and fidelity indicate operational promise, not long-term sustainability. Most residents completed the introductory schedule, observation exceeded 20% in each intervention home, and routine logs were nearly complete. Interviews nevertheless identified cleaning, storage, documentation, turnover, and responsibility as implementation risks. Staff valued role-play but wanted coaching for conflict and over-attachment. These findings suggest that a future model may need a site champion, competency reassessment, audit and feedback, and protected documentation time rather than establishing those components as universal requirements.

The safety profile was acceptable for an exploratory low-risk psychosocial intervention, but not event-free. Six events were considered possibly or probably related, and routine records documented refusal, distress, and possessiveness or conflict. No serious intervention-related event occurred. Routine engagement episodes could not be linked uniquely to adverse-event adjudications; therefore, the reported frequencies should not be interpreted as incidence rates. Future studies should distinguish expected transient discomfort, protocol-defined adverse events, serious adverse events, and ethical deviations; link them to exposure time; and capture whether reintroduction was attempted. A data-monitoring threshold may be unnecessary for a small pilot but should be reconsidered for a larger multicenter trial.

This study has several strengths. It evaluated a culturally and ethically sensitive intervention in an under-studied Chinese residential-care context; used cluster allocation to limit contamination; maintained a clear hierarchy separating medication, behavioral, functional, and contextual outcomes; and combined clinical, process, fidelity, safety, and qualitative evidence. Outcome components and standardized medication doses were calculated consistently from prespecified variables, and missing medication information was not classified as non-exposure. Cluster-level and missing-data sensitivity analyses were compared directionally with the marginal models. Qualitative analysis represented favorable, neutral, mixed, and unfavorable accounts, and selected quotations were source-checked against their Mandarin transcripts.

The limitations are substantial. First, six randomized homes provide too few independent units to yield stable estimates. The Mancl–DeRouen correction and a four-degree-of-freedom t-distribution reduce but do not eliminate bias, and the fully iterated jackknife was numerically invalid. Second, the constrained randomization candidate set was unavailable; the exact analysis over all 20 allocations was therefore a sensitivity analysis rather than the prespecified test. Third, residents and deliverers could not be blinded, and assessment-level unblinding flags did not reconcile with the deviation log. Fourth, the comparator did not control for staff attention, structured interaction, or additional activity, so the package effect cannot be separated from attention. Fifth, medication outcomes were zero-inflated, some records were missing, prescribing was not controlled for by the intervention, and pre-existing medication-review frequency was completely confounded with arm. Sixth, the study-specific traditional Chinese medicine score was unvalidated. Seventh, qualitative interviews were limited to intervention homes and may overrepresent engaged participants or staff willing to discuss the program. Prospective documentation of coder profiles, positionality, and formal consensus procedures was incomplete, which limits assessment of analytic reflexivity and dependability. Eighth, the baseline NPI-12 strata were *post hoc* pragmatic score bands rather than validated severity categories; their small cell sizes and susceptibility to regression to the mean preclude subgroup efficacy claims.

The findings have implications for long-term-care practice and public health. Consistent with person-centered and ethical accounts of doll therapy (9, 16, 17), doll therapy should be considered neither a universal activity nor a replacement for relational staffing. It may be an optional component of dementia care for residents who show interest in or comfort with it. The present findings support non-deceptive explanation, continuous assent, immediate stopping after refusal, family communication, infection-control procedures, staff competency, and access to alternatives within this care package. For medication stewardship, prior multicomponent trials indicate that behavioral interventions are more likely to affect prescribing when coupled with explicit review pathways (37–39). At the system level, the study illustrates the importance of evaluating implementation and ethics alongside mean clinical outcomes when introducing low-cost psychosocial care in rapidly expanding long-term care sectors.

A definitive trial would require substantially more nursing homes, detailed documentation of allocation procedures to support exact inference, an active attention-control condition, blinded outcome assessment where feasible, prospective progression criteria, and a prespecified multiplicity strategy. Given the instability of analyses with very few clusters (32), randomization should be stratified or constrained on key site characteristics, with the candidate allocation set and balance criterion documented prospectively. The present findings further support retaining individual stopping rules and independently observed fidelity while adding ethical-fidelity items and observer-level data. Outcomes should prioritize a proximal behavioral measure, include engagement and quality-of-interaction measures, and treat medication reduction as a linked service-delivery endpoint supported by a formal review process.

## Conclusion

In this exploratory mixed-methods cluster-randomized trial, a staff-mediated doll-therapy care package was feasible to deliver in Chinese nursing homes with high session exposure and fidelity. NPI-12 results provided a preliminary behavioral signal that was directionally supported across sensitivity analyses, while medication-dose effects were imprecise and functional improvement was not sustained at 12 weeks. Acceptability depended on individualized response, staff skill, non-deceptive communication, family engagement, and immediate respect for refusal. These results justify a larger, better-powered cluster trial of the full care package, but do not establish a definitive clinical or medication-reduction effect.

## Data Availability

The raw data supporting the conclusions of this article will be made available by the authors, without undue reservation.
